# Improving Mood Promoting Access to Collaborative Treatment (IMPACT) Program in Geriatrics Primary Care

**DOI:** 10.1093/geroni/igab046.1085

**Published:** 2021-12-17

**Authors:** Shahla Baharlou, Gregory Hinrichsen, Lizette Munoz, Katherine Currey, Sheila Barton, Fay Kahan, Blair MacKenzie, Laili Soleimani

**Affiliations:** 1 Icahn School of Medicine at Mount Sinai, Icahn School of Medicine at Mount Sinai, New York, United States; 2 Icahn School of Medicine at Mount Sinai, New York, New York, United States; 3 Mount Sinai Health System, New York, New York, United States

## Abstract

Improving Mood Promoting Access to Collaborative Treatment (IMPACT) is a well-established model for the treatment of depression in primary care. The COVID pandemic has caused increased distress and depression among the older patients in our New York City geriatric practice. This paper describes the establishment of a virtual IMPACT model during the pandemic in which IMPACT services have been provided via telephone. This effort was a multidisciplinary collaboration among geriatric medicine, geriatric psychiatry, social work, and geropsychology. Our IMPACT program uses a brief form of Interpersonal Psychotherapy (IPT) for depression as the psychosocial component instead of Problem Solving Treatment. Delivery of IMPACT by telephone appears to have enhanced engagement and sustained involvement in the program compared with prior efforts to deliver it by in-person meetings. IPT as a psychosocial modality was well-received by patients. To date, treatment outcomes have been favorable and will be reported in this presentation.

